# Bootstrapping Knowledge Graphs From Images and Text

**DOI:** 10.3389/fnbot.2019.00093

**Published:** 2019-11-12

**Authors:** Jiayuan Mao, Yuan Yao, Stefan Heinrich, Tobias Hinz, Cornelius Weber, Stefan Wermter, Zhiyuan Liu, Maosong Sun

**Affiliations:** ^1^Natural Language Processing Lab, Department of Computer Science and Technology, Tsinghua University, Beijing, China; ^2^Knowledge Technology Group, Department of Informatics, University of Hamburg, Hamburg, Germany

**Keywords:** relation learning, relation prediction, information extraction, knowledge graphs, inductive logic programming

## Abstract

The problem of generating structured Knowledge Graphs (KGs) is difficult and open but relevant to a range of tasks related to decision making and information augmentation. A promising approach is to study generating KGs as a relational representation of inputs (e.g., textual paragraphs or natural images), where nodes represent the entities and edges represent the relations. This procedure is naturally a mixture of two phases: extracting primary relations from input, and completing the KG with reasoning. In this paper, we propose a hybrid KG builder that combines these two phases in a unified framework and generates KGs from scratch. Specifically, we employ a neural relation extractor resolving primary relations from input and a differentiable inductive logic programming (ILP) model that iteratively completes the KG. We evaluate our framework in both textual and visual domains and achieve comparable performance on relation extraction datasets based on Wikidata and the Visual Genome. The framework surpasses neural baselines by a noticeable gap in reasoning out dense KGs and overall performs particularly well for rare relations.

## 1. Introduction

For human infants, it is seemingly easy to learn to reason about the relation between any two objects. Infants show this capability because they learn to understand the world, and they acquire language by integrating cross-modal information. In particular, they do not only learn referents in language by statistically matching words with occurrences of objects in the environment, but also begin to understand the characteristics and affordances of the objects. AI systems, however, are usually developed based on single modalities or tasks with limited access to the context. Since a crucial aspect of current AI systems is to learn appropriate representations for designated tasks, it seems particularly important to reflect cross-modal learning also in learning these representations. For learning relational representations, let's consider the following example. In *The Little Match Girl*'s dream, Hans Christian Andersen wrote:

On the table was spread a snow-white tablecloth; upon it was a splendid porcelain service, and the roast goose was steaming famously with its stuffing of apple and dried plums.

**Figure 1:**
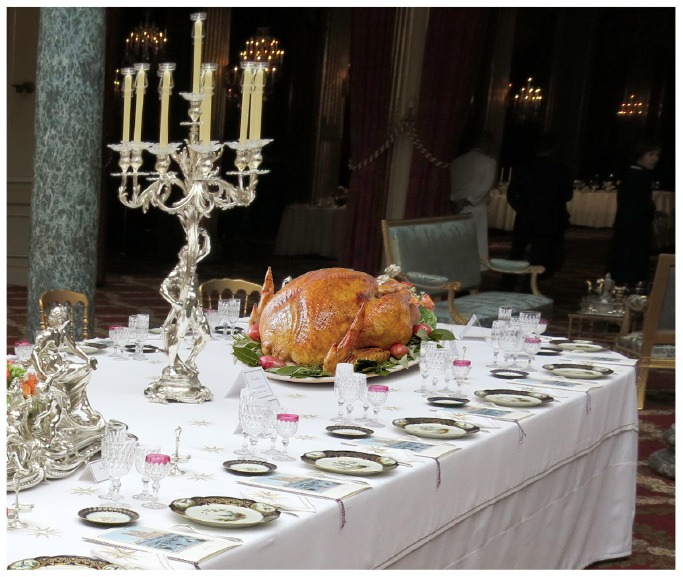
The scene^1^.

**Figure 2:**
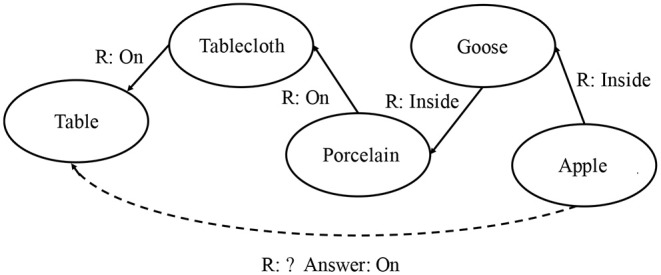
Resulting semantic graph of the scene in Andersen's *The Little Match Girl*'s dream.

From either the text, or an image capturing the scene (Figure 1), it is effortless to conclude that Tablecloth is On the Table, while the Apple is Inside a Goose which is On a porcelain dish put on the Tablecloth. Now, let us ask another question: what is the relation between Apple and Table? Figure 2 indicates the reasoning chain.

For us human beings this requires little reasoning efforts, even infants can get the answer On. However, current computational architectures barely support it, not even for restricted purposes.

The process above can be more broadly described as relation extraction, which is to determine the relationship between objects (entities) that appear in a textual paragraph or in a visual scene. We focus on the problem of conditional relation extraction, which generates a graph regarding a specific paragraph or an image, with each edge representing a relation instance *(subject, object, relation)* such as (Apple,Table,On). We call the resulting graph “Semantic Graph” for textual paragraphs (Sorokin and Gurevych, 2017), and “Scene Graph” for visual images (Xu et al., 2017), respectively. By bringing together textual and visual relation extraction, we are particularly interested in dependencies between both modalities and how synergies lead to more robust representation learning. Relation extraction additionally has many potential applications, including question answering (Xu et al., 2016), fact checking (Vlachos and Riedel, 2014), word sense disambiguation (Okamoto and Ishizaki, 2007), and document summarization (Hachey, 2009).

In most recent literature (Sorokin and Gurevych, 2017; Xu et al., 2017), the generation of knowledge graphs (KGs) is decomposed into two phases: (1) detecting the entities (or objects) as nodes, and (2) extracting relations between entities as edges. The first phase can be reduced to a Named Entity Recognition for textual paragraphs (Lample et al., 2016) or Object Detection for images (Ren et al., 2015). Usually, the more challenging part receiving more attention is how to determine the relations between entities and is usually cast as a classification problem. A critical difference between relation extraction and typical classification problems [e.g., image classification (Krizhevsky et al., 2012) or natural language inference (Bowman et al., 2015)] lies in the existence of dependencies between relation instances. That is, two entities, even when separated spatially, may form a relation when they are both related to one or more other entities (in the above case, Apple and Table both interact with Tablecloth, Dish, etc.).

In order to deal with this challenge, we extend the second step—extracting relations between entities—by applying a set of differentiable logic rules to extract further relations based on the current KG. We note that the process of predicting unknown relations based on the current (incomplete) KG shares some commonalities with knowledge graph completion (KGC). The reasoning module of our model predicts unknown relations by applying first-order logic rules, which can be naturally replaced with previous KGC methods. However, most of the previous approaches for KGC make the completion by learning representations of entities and relations and viewing relations as translations between entities (Bordes et al., 2013; Wang et al., 2014; Ji et al., 2015; Lin et al., 2015). In contrast, we apply logical, rule-based reasoning in order to find unknown relations, similarly to Yang et al. (2017). However, while the model by Yang et al. (2017) works only for global KGC our approach finds unknown relations in a contextual manner.

Informally, given the set of entities, determining the relation between them can be viewed as a mixture of two sub-tasks: (1) extracting primary relations from the input and (2) completing the KG with reasoning. Primary relations are mostly literal ones such as, in the match girl's example (Tablecloth, Table, On). The completion of the KG, on the other hand, requires reasoning over these primary relations and resolving the dependencies or correlations.

**Figure 3:**
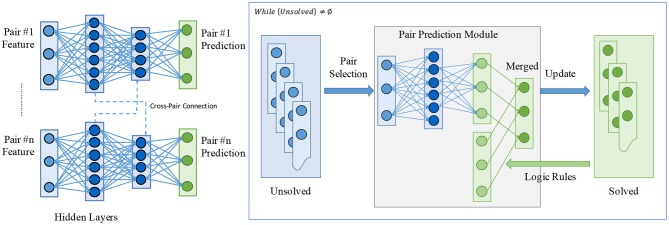
An illustration on the comparison between typical neural network-based relation extractors and the proposed hybrid relation extractor. (Left) A general framework for typical neural network-based relation extractors. Dependencies or correlations between relation instances are modeled by viewing other relations as the “context.” Dashed connections denote the cross-pair connections, which may involve an attention mechanism (Sorokin and Gurevych, 2017) or message passing (Xu et al., 2017). (Right) The proposed hybrid relation extractor (HRE) working in an iterative manner. With a pair selector collaborating with a predictor, it naturally resolves the dependencies or correlations between relation instances. The pair prediction module works with a relation induction model based on explanatory logical rules. See section 3 for details.

In the past, methods based on neural networks have been shown to be successful on a large range of tasks in various fields (LeCun et al., 2015). Particular approaches try to resolve the dependencies between relation instances by modeling other relations as the “context” of specific pair entities. A number of researches have been done in this direction, such as attention-based encoding (Sorokin and Gurevych, 2017) or graph-based message passing (Xu et al., 2017). Figure 3, left, shows a general framework for neural network-based relation extractors. There are multiple drawbacks of such contextual encoders:

1. From a systematical point of view, although neural networks are Turing-complete (Siegelmann and Sontag, 1995), and can be wired to mimic any computer circuit, in practice, they are more suitable for processing associations rather than rules. For example, starting from (Fodor and Pylyshyn, 1988), there has been a long-lasting debate over the problem of systematicity (such as understanding recursive systems) in such connectionist models (Fodor and McLaughlin, 1990; Hadley, 1994; Jansen and Watter, 2012). In the case of relation extraction, reasoning is usually performed in a chained, or recursive way (e.g., consider the relation between Apple and Table in the match girl's example), which the contextual autoencoders do not reproduce.
2. From an implementational point of view, relation extraction requires the processing of high-order relational data and quantifiers. For example, to apply the transitivity: (Tablecloth, Table, On) ∧ (Porcelain, Tablecloth, On) ⇒ (Porcelain, Table, On), we need to consider the relation among a triple of symbols (Table, Tablecloth, Porcelain). This is beyond the scope of typical graph-structured neural networks (Kipf and Welling, 2016).
3. In most datasets, the distribution of relations is uneven and has a long tail of rarely occurring relations between specific objects. Approaches based purely on neural networks have problems to learn these rare object-relation triplets due to the limited number of occurrences in the training data and can often not generalize them to objects not seen during training. In contrast, by using inductive reasoning we can incorporate previous knowledge about the characteristics of relations into the KG generation process to help extract rare relations and to increase generalizability. Inductive reasoning can be especially powerful for transitive relations [e.g., geometric (left, right) or possessive (is-part-of) relations] which make up most of the relations in many datasets (Zellers et al., 2018). Many datasets actually miss labels for relations that occur in the data due to incomplete labeling (Wan et al., 2018), which means that models that are trained purely on the (labeled) data do not learn about these relations.
4. Recent work also indicates that purely neural approaches do not generalize learned relations (e.g., spatial ones), at least not in the vision domain (Kim et al., 2018; Bahdanau et al., 2019). This means that simple learned relations such as “left of” do not usually generalize to novel object combinations. Currently, the only way for these neural architectures to generalize is by using a perfect model architecture specifically tailored for the domain (dataset), such as optimally constructed Neural Module Networks (Andreas et al., 2016). This is challenging because we need perfect knowledge about the data and the relations for this, which is not possible for real-world datasets. Our hypothesis is that logical reasoning is implicitly better suited to handle the generalization of relations since it is a symbolic approach and models the relations independently of the objects they refer to.
5. Another challenge is that in the vision domain the processing is usually done with convolutional neural networks (CNNs), which only perform local pixel-level reasoning (Chen et al., 2018), making it difficult to extract relations between objects that are far apart. However, especially for transitive relations (which can be extracted with logic rules), large distances (in pixel-space) between objects are very common.

To address the above issues, in this paper we propose a hybrid KG builder that combines two procedures into a unified framework and generates KGs from scratch using visual or textual information. As described in section 3 in more detail, we employ (1) a neural relation extractor detecting primary relations from the input and (2) a differentiable inductive logic programming (Muggleton, 1991) model that iteratively completes the KG. We suggest the neural relation extractor as a key element because relations between entities are usually tightly interwoven within high dimensionality and neural networks are particularly good in learning distributed representations. The programmable logic induction system element, on the other hand, is especially strong in extracting the structure of facts from natural language and images. In this framework, relations between entities are detected by the joint effort of the neural module as well as the logic module.

Through extensive experiments in section 4, we compare our framework against strong relation extraction baselines in both textual and visual domains, on a Wikidata-based dataset and the Visual Genome dataset, respectively. Empirical results show the superiority and flexibility of our proposed method. Moreover, we show a significant gain over baselines and other prior works in a subset of the database that contains dense graphs^2^, i.e., a higher than average number of relations per entity pair. We discuss related works in section 2 and conclude in section 5.

## 2. Related Work

Relation extraction is an important task and necessary to obtain a detailed understanding of texts or images. In the following we first describe current approaches for relation extraction from textual data, before continuing to describe relation extraction from images.

### 2.1. Relation Extraction From Texts

Relation extraction has been widely used to obtain structured knowledge from plain text. The resulting structured relational facts are crucial to understanding large-scale corpora and can be utilized to automatically complete missing facts in KGs. Early neural relation extraction methods generally attempted a supervised paradigm (Zeng et al., 2014; Nguyen and Grishman, 2015; Santos et al., 2015) and heavily rely on human-labeled datasets. However, the annotation of these datasets is labor-intensive and time-consuming. Recent relation extraction methods address the problem by creating large-scale training data via distant supervision. However, the assumption of distant supervision is very strong and often introduces noise. Much work has been invested in order to alleviate the wrong-labeling problem in distant supervision and to extract global relations between two entities from multiple supporting sentences (Riedel et al., 2010; Zeng et al., 2015; Lin et al., 2017; Feng et al., 2018; Qin et al., 2018). Recently many approaches also explore the extraction of relations between entities on the sentence level in rich context (Sorokin and Gurevych, 2017; Zeng et al., 2017; Christopoulou et al., 2019; Zhu et al., 2019).

Mintz et al. (2009) propose distant supervision to automatically generate a large-scale dataset for relation extraction by aligning plain text with knowledge graphs. The assumption of distant supervision is that all sentences containing an entity will express the corresponding relation in KGs. Zeng et al. (2015) further formulate distantly supervised relation extraction as a multi-instance learning problem, where instance bags consist of multiple sentences containing an entity pair, and take the uncertainty of instance label into consideration by selecting the most confident supporting instance for relation prediction. Lin et al. (2017) propose to obtain bag representations by semantic composition of instances, where instance weights are determined by selective attention. Feng et al. (2018) propose to filter false positive relation instances via reinforcement learning. Qin et al. (2018) propose an adversarial framework that jointly learns a generator and discriminator to distinguish false positive relation instances from distant supervision.

Sorokin and Gurevych (2017) identify sentence-level relation between entity pairs in a rich context. They predict relations between each entity pairs by considering all other possible entity pairs in the same sentence as context and modeling the correlation of relations via attention mechanism. Christopoulou et al. (2019) model the context of an entity pair by iteratively aggregating walk paths between the target entity pair on the graph, and achieve comparable results without using external linguistic tools. Zhu et al. (2019) model implicit reasoning via message passing among context entity pairs. In this work, we also focus on extracting sentence-level relations. A crucial difference is that we extract relations within a sentence or paragraph sequentially to explicitly model the relation reasoning structure. Zeng et al. (2017) explicitly use a special first-order logic rule to model the dependencies of relations within a sentence. A crucial distinction of our model is that we are capable of modeling general and also long reasoning chains by recursively applying rules.

### 2.2. Relation Extraction From Images

In order to understand and reason about the context of an image we need not only information about objects within the scene, but also about relations between these objects. Therefore, extracting the relations between objects (e.g., in/on/under, support, etc.) yields a better scene understanding compared to just recognizing objects and their individual properties (Elliott and de Vries, 2015). While relations can be predicted pair-wise (Chao et al., 2015; Ramanathan et al., 2015), most current work focuses on the generation of a directed graph generally referred to as scene graph (Johnson et al., 2015; Xu et al., 2017; Zhang et al., 2017). Scene graphs are a way of representing the context of an image in a structured way to improve the performance of tasks such as visual question answering or image retrieval. Existing scene graph generators usually extend an object detection framework that first detects bounding boxes for objects, then extracts visual features and classifies objects inside bounding boxes, and finally predicts relations between objects in a parallel manner. One of the challenges is that the number of possible relations grows exponentially with the number of objects in an image. This makes it computationally challenging to evaluate all possible relations. Therefore, many approaches work on ways to prune unlikely relations from the graph or to only focus on the most probable relations from the beginning.

Li et al. (2017) combine three tasks—object detection, scene graph generation, and region captioning—and show that learning all three tasks at once leads to an overall better performance since learned features can be shared across tasks. Xu et al. (2017) propose an end-to-end trainable approach for creating an image-grounded scene graph that consists of object categories, bounding boxes for the individual objects, and relationships between pairs of objects by iteratively refining its predictions. Liang et al. (2017) perform prediction together with a traversal of the graph, essentially in a sequential manner. However, it takes only the last two prediction results into account and thus is unable to perform general logic inductions based on a partial inference result.

Li and Gupta (2018) learn to transform 2D image representations into a graph representation where the nodes represent image regions and edges model similarity between these image regions while Chen et al. (2018) introduce a graph structure specifically to facilitate reasoning between regions that are far apart in the image. Yang et al. (2018) make the scene graph generation more tractable and efficient by using a relation proposal network that identifies likely edges in the scene graph and a Graph Convolutional Network to update objects and their relationships based on the objects' neighbors. Woo et al. (2018) propose a relational embedding module to jointly represent connections among all objects instead of focusing on objects in isolation.

Related to our approach, Wan et al. (2018) work on completing existing scene graphs given an image and a corresponding scene graph. However, they do not use logic reasoning, but instead, use a neural network to extract unidentified relations between existing nodes in the scene graph to obtain improved scene graphs with more accurate relations. The approach, however, is still completely data-driven and, as such, it is not clear how it handles the long tail of sparsely occurring relations and how it generalizes to novel object-relation triplets.

Zellers et al. (2018) observe that object labels are highly predictive of relation labels (but not vice versa) and use this insight to develop both a new baseline and a network that takes this into consideration by staging bounding box predictions, object identities (in the bounding boxes), and relations in a hierarchical manner. Chen et al. (2019) show that using the knowledge about correlations between objects and associated relations can be explicitly represented in a KG. A novel routing network then facilitates scene graph generation by using prior statistical knowledge about the interplay of objects and relations.

Gu et al. (2019) incorporate commonsense knowledge into the generation process of a scene graph by using an external KG while Qi et al. (2019) use linguistic knowledge to improve the performance on detecting semantic relations by using a semantic transformation module to map visual features and word embeddings into a common semantic space. So far, most work on extracting scene graphs from images is based purely on data-driven learning with neural networks. This creates challenges in scalability (especially for images with many objects) and suffers from the long tail of relations in the training data, which is difficult to learn for neural network-based approaches. Additionally, it is not clear whether these approaches are able to generalize learned relations to novel settings. In contrast, our approach combines data-driven neural networks with a differentiable model that applies logic rules for relation extraction. This enables us to insert prior knowledge about certain relations (e.g., transitivity) into our model which can help with generalizability (since relations are now decoupled from the objects), scalability (we can efficiently evaluate the learned rules), and the long tail of relations in the training data (once a rule encodes one of these relations we can easily apply it to other objects, too).

## 3. Methods

We build our hybrid relation extractor (HRE) by combining a neural relation extractor detecting primary relations from inputs and an inductive logic-based model that iteratively completes the KG. Illustrated in Figure 3, right, the framework works in an iterative manner and detects the relations by the joint work of the neural module and the logic module. As discussed in the above section, there are two major challenges for modeling the relation reasoning:

1. Chaining or recursions. We resolve the dependencies among relations by iteratively detecting edges. Specifically, we propose to use a pair selector working jointly with the relation predictor.
2. High ordering and quantifiers. We model relation reasoning with a differentiable inductive logic programming (ILP) model (Muggleton, 1991). The model discovers probabilistic rules from examples by inductive reasoning.

In the rest of the paper we write (*subject, object, relation*) to denote a specific relational triplet, while *rel*(*object, subject*) is used to refer to the distribution over relations for an entity pair, and *rel*(*object, subject*)_*i*_ is the probability of relation i to be true. We now begin the introduction of the model with an overview.

### 3.1. Overview of the Framework

We build our framework on the top of the extracted entities by either named entity recognition algorithms (Lample et al., 2016) or object detectors (Ren et al., 2015). Specifically, for each paragraph or image, we first use entity detectors to find all of the entities and localize them. In the textual paragraph case, we match all tokens and phrases in the paragraphs with the entities appeared in the Wikidata dataset. In the visual image case, we employ Faster-RCNN (Ren et al., 2015), a modern CNN-based object detector to find all entities and determine their class labels. For a detailed analysis of the dataset and the pre-processing, please refer to section 4.

After the pre-processing, the relation extractor takes all possible entity pairs as input, and assigns proper relations to each pair. As shown in Figure 3, the HRE contains two units, a pair selector and a relation predictor, and runs in an *iterative* way. At each time step, the pair selector takes a look at all pairs $P^{-}={\left(s_{i},o_{i}\right)}_{i=0}^{k_{-}}$ of *(subject, object)* that have not been associated with a relation and chooses the next pair of entities p^*^ = (s^*^, o^*^) whose relation is to be determined. The relation predictor utilizes the information contained in all pairs $P^{+}={\left(s_{i},o_{i},r\right)}_{i=0}^{k_{+}}$ whose relations have been determined and the contextual information (from raw texts or images) of the pair p^*^ to make the prediction on the relation. The prediction result is then added to *P*^+^ and benefits future predictions.

The pair selector and relation predictor work jointly and focus on different sub-problems of the task. The predictor's objective is to make use of the relations that have already been determined in order to make a valid prediction for the next entity pair. The selector, on the other hand, works as the predictor's collaborator with the goal to figure out the next relation which should be determined. Ideally, the choice p^*^ made by the selector should satisfy the condition that all relations that will affect the predictor's prediction on p^*^ should be sent to the predictor ahead of p^*^.

### 3.2. Relation Predictor

The relation predictor is composed of two modules: a neural module predicting the relations N between entities based on the given context (i.e., a textual paragraph or a visual image) and a differentiable inductive logic module L performing reasoning on *P*^+^ (the set of pairs whose relations have already been determined). Both modules predict the relation between a pair of objects s^*^ and o^*^ individually as $rel_{N}\left(s^{*},o^{*}\right)$ and $rel_{L}\left(s^{*},o^{*}\right)$. These predictions are classifications over a categorical distribution of all relations: $rel_{N}{\left(s^{*},o^{*}\right)}_{i}=\mathrm{Pr}\left[rel_{N}\left(s^{*},o^{*}\right)=i\right]$ and $rel_{L}{\left(s^{*},o^{*}\right)}_{i}=\mathrm{Pr}\left[rel_{L}\left(s^{*},o^{*}\right)=i\right]$. The output prediction for the pair p^*^ is a mixture^3^ of the two individual predictions:

$$\begin{array}{l} rel{\left(s^{*},o^{*}\right)}_{i}\propto rel_{N}{\left(s^{*},o^{*}\right)}_{i}\times rel_{L}{\left(s^{*},o^{*}\right)}_{i}. \end{array}$$

The neural relation extractor *rel*_*N*_ is domain-specific. We leave the implementation of *rel*_*N*_ to the experiment section (section 4). In real-world applications, this module can be replaced by any compatible implementation. In the following, we present our model L for KG reasoning, which is a differentiable variant of inductive logic programming (Muggleton, 1991).

We design a programmable module for KG reasoning, which is highly motivated by previous works on inductive logic programming (ILP) (Muggleton, 1991) and its modern extensions (Kersting et al., 2000; Richardson and Domingos, 2006; Kimmig et al., 2012). ILP focuses on the problem of how to discover rules from known facts and applies them to deduce unknown facts.

To get an intuitive idea on how ILP works, we take *The Little Match Girl*'s dream as an example. We want a model that is able to perform logic deduction:

$$\begin{array}{l} (\text{Tablecloth},\text{ Table},\text{ On})\wedge (\text{Porcelain},\\ \text{Tablecloth},\text{ On})\;\Rightarrow \;(\text{Porcelain},\text{ Table},\text{ On}). \end{array}$$

This logic rule can be generally written as a *definite clause*:

(x,y,On)∧(y,z,On)⇒(x,z,On),

where *x, y*, and *z* are variables that can be replaced (*grounded*) by any entities such as Tablecloth, Table, and Porcelain.

ILP is a general programming framework, which provides a higher level of abstraction on logic rules. For example, the above logic rule can be derived (*instantiated*) by the following *meta-rule* in ILP:

$$\begin{array}{r} r_{1}=rel\left(s^{*},x\right)\in P^{+}\wedge \;r_{2}=rel\left(x,o^{*}\right)\in P^{+}\Rightarrow \;rel\left(s^{*},o^{*}\right)=r_{3}.\left(1\right) \end{array}$$

In the instantiation of the meta-rule, *r*_1_, *r*_2_, and *r*_3_ will be instantiated as (On, On, On). Another possible instantiation can be (Inside, On, On). Intuitively, the entity triple (s^*^, x, o^*^) essentially forms a “relation triangle,” and we use two of the edges which we already know — (s^*^, *x*) and (x, o^*^) — to determine the last edge (s^*^, o^*^).

Practically, the underlying logic is a probabilistic logic. That is, we will say

$$\begin{array}{l} \mathrm{Pr}[r_{3}=rel(s^{*},o^{*})]\propto \mathrm{Pr}[r_{1}=rel(s^{*},x)]\;\times \;\mathrm{Pr}[r_{2}=rel(x,o^{*})]\\ \;\times confidence(r_{1},r_{2},r_{3}), \end{array}$$

where *confidence*(*r*_1_, *r*_2_, *r*_3_) is the confidence (a floating number) associated with the applied rule. We implement the logic induction programming in a differentiable manner. Unless explicitly specified, all rules are derived from Equation (1) in this paper. During inference, relations between all entity pairs are predicted. Thus, a long reasoning chain (e.g., Table, Tablecloth, Dish, Goose in *The Little Match Girl*'s dream) can be resolved by multiple primitive logic deduction steps. In this case, the simple “triangular” logic rule (Equation 1) is sufficient to resolve a long reasoning chain.

Given a set of rules R instantiated from a pre-programmed set of meta-rules, we enumerate all rules and compute the final prediction from inductive logic module L as:

$$\begin{array}{l} rel_{L}{\left(s^{*},\;o^{*}\right)}_{i}\;\propto \;\underset{rule\in ℛ}{\max}\;rule{\left(s^{*},o^{*};P^{-}\right)}_{i}\\ \;\propto \;\underset{j,k,x}{\max}\left(rel{\left(s^{*},\;x\right)}_{j}\;\times \;rel{\left(x,\;o^{*}\right)}_{k}\;\times \;confidence(j,k,i)\right) \end{array}$$

The tensor *confidence*, as the representation of logic rules, is optimized through back-propagation during the training.

Given a set of relation instances, the aforementioned logic rule is just one choice to perform induction. In practice, one can design own rules based on the characteristic of the dataset or the underlying application. We show in the experiments section that the system is compatible with other rules and yields different results.

### 3.3. Pair Selector

The pair selector works together with the relation prediction module and chooses subject-object pairs for prediction. At each time step, the pair selector takes a look at all relation pairs in $P^{-}={\left(s_{i},o_{i}\right)}_{i=0}^{k_{-}}$ whose relations have not been determined and outputs an index *i* ∈ [*k*_−_] = {0, 1, ⋯*k*_−_} as the index for the entity pair whose relation will be added to *P*^+^ by the predictor in this time step.

We implement the pair selector as a greedy selector which always chooses the entity pair from *P*^−^ to be added to *P*^+^ as the entity pair of which the relation predictor is most confident in its prediction. The relation predictor's output probability Pr(*r* = *rel*(s^*^, o^*^)) (section 3.2) can be interpreted as its confidence for assigning the relation *r* to the pair (s^*^, o^*^):

$$\begin{array}{l} conf\left(s^{*},o^{*}\right)=\underset{r}{\max}\;\mathrm{Pr}\left(r=rel\left(s^{*},o^{*}\right)\right). \end{array}$$

Thus, in order to choose the pair of which the relation predictor is most confident, the pair selector chooses *i* such that:

$$\begin{array}{l} i=\underset{i}{\max}conf\left(s_{i},o_{i}\right). \end{array}$$

## 4. Experiments and Results

We evaluate our model on tasks for two modalities: textual and visual relation extraction. Our aim is to study how the hybrid relation extraction is affected by different encoding and how it scales for different complexity. Our experiments show that it outperforms other approaches by a noticeable gap when dealing with dense entity graphs.

### 4.1. Textual Relation Extraction

#### 4.1.1. Entity Pair Encoding in Text

**Figure 4:**
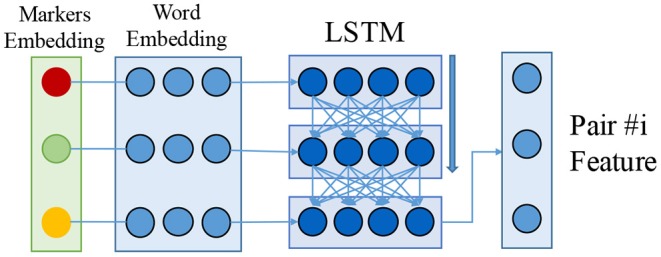
Encoder for textual entity pairs. We use the concatenation of marker embedding and word embedding with an LSTM model (Hochreiter and Schmidhuber, 1997; Greff et al., 2016) to encode the feature.

Recall that we need to predict a relation for each possible entity pair. For the textual relation extraction task, we encode the features of an entity pair following Sorokin and Gurevych (2017) as shown in Figure 4. First, we pre-process the sentence and run named-entity-recognition to find all relevant entities. We then add an extra embedding as a marker indicating all appearances of the given head (subject, with *e*_*s*_) and tail (object, with *e*_*o*_) of the entity pair. All other context symbols are marked with *e*_*c*_. The embeddings {*e*_*s*_, *e*_*o*_, *e*_*c*_} are initialized randomly and jointly optimized with the model.

The marker embedding is concatenated with the word embedding (Pennington et al., 2014) and passed to a bi-directional LSTM (Hochreiter and Schmidhuber, 1997; Graves and Schmidhuber, 2005; Greff et al., 2016). We use a standard bi-directional LSTM with one layer, 256 LSTM units, the TANH activation function, and 0.5 dropout rate Srivastava et al. (2014). The final outputs of the LSTM of both forward and backward passes are concatenated as the final encoding for this entity pair. We apply a two-layer multi-layer perceptron followed by a softmax layer on the feature for neural relation extraction: *rel*_*N*_. This process is repeated for each possible entity pair in the sentence, i.e., *n* × (*n*−1) times for a sentence with *n* entity pairs.

#### 4.1.2. Data Generation With Distant Supervision

We introduce a new dataset generated from Wikidata (Vrandečić and Krötzsch, 2014) to evaluate our framework on the task of textual relation extraction. Wikidata is a KG which stores knowledge as structured triplets, (e.g., Earth, Mount Everest, highest point). We align Wikidata with English Wikipedia articles via distant supervision (Mintz et al., 2009; Zeng et al., 2015; Sorokin and Gurevych, 2017). We select the 86 most frequent properties (relations) to form the property set.

We generate paragraphs by concatenating two sentences which are chosen from the same article. The selected sentences should share at least one common entity. This partially alleviates the sparsity of relations. For entity pairs without relation, we manually mark their relation as N/A (a special relation). We also filter out paragraphs that contain fewer than 2 positive relation instances. Following the setting of previous work (Lu et al., 2016; Xu et al., 2017), in our experiments, we randomly split the dataset into training and test sets, and tune the hyper-parameters of all models on the test set. We manually evaluate 500 sentences from the test set and find 83.2% of them are correctly labeled with distant supervision. Table 1 shows the statistics of our dataset.

**Table 1:** Statistics of the dataset generated from Wikidata.

	**#Sent**	**#Fact**	**#Avg ent**.	**#Avg pos. rel**.
Train	124,212	70,598	5.51	2.47
Test	31,054	29,148	5.56	2.33

*#Avg ent. stands for the average number of entities per paragraph, while #Avg pos. rel. refers to the average number of positive relations per paragraph*.

The dataset generated from Wikidata is very sparse with respect to relation instances: each sentence contains only 2.7 relation instances on average and the fraction of relation instances over the entity pairs is less than 0.12. To better focus on evaluating the reasoning ability of our model, we select a dense test set where semantic graphs can be deduced^4^. Within the dense subset reasoning chains are substantially more common which requires the model to perform both primary relation detection and relation reasoning. The dense test set covers ~ 2% of the whole dataset. We adopt the precision-recall curve, a widely used metric in textual relation extraction. The *k*-th point in the curve is computed by the precision and recall of the top *k* confident predictions. We also report the F_1_ score (Goutte and Gaussier, 2005), which is computed by the harmonic average of the precision and recall of the most confident predictions of each entity pair. The special relation N/A does not affect recall but only precision.

**Figure 5:**
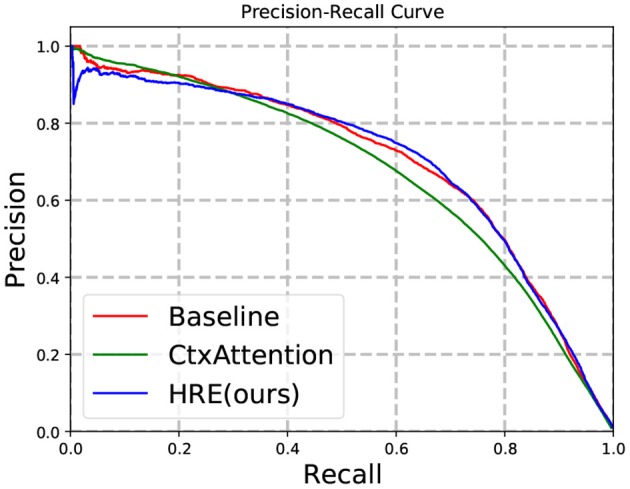
Precision-recall on the dense test set.

We train our model on the entire training set and evaluate the performance on the dense test set. In Figure 5 and Table 2, experimental results show that our model significantly outperforms baseline methods (with only *rel*_*N*_) on the dense test set. To further zoom in, we compare the recall score of all frameworks under a moderate precision (e.g., 0.8) in Table 3. The strong baseline is identical to, and is a re-implementation of, the model used by Sorokin and Gurevych (2017).

**Table 2:** F1 scores on the dense test set.

**Model**	**Micro F1**		
	**P**	**R**	**F1**
Baseline	0.75	0.56	0.640
Ctx Attention	**0.77**	0.52	0.621
HRE	0.72	**0.63**	**0.675**

*The best results are highlighted in bold*.

**Table 3:** Recall at different precision levels on the dense test set.

	**Baseline**	**Ctx Attention**	**HRE**
R@0.60	**0.741**	0.674	0.740
R@0.70	0.633	0.574	**0.661**
R@0.80	0.488	0.444	**0.505**

*The best results are highlighted in bold*.

**Figure 6:**
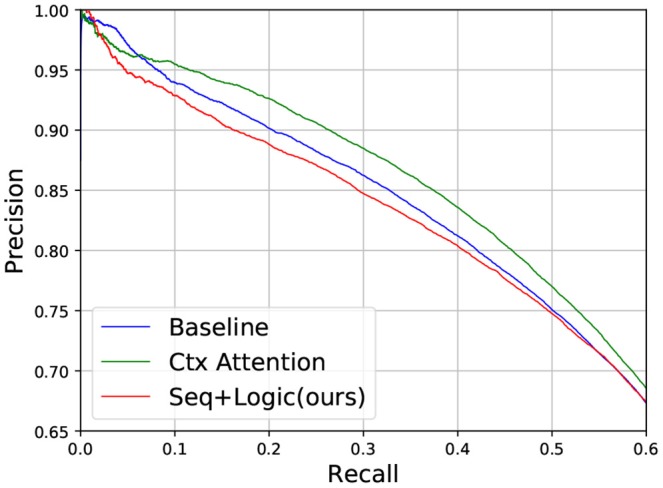
Precision-recall on the entire test set.

We also show a comparable result on the entire test set (Figure 6 and Table 4). In this case, logic deduction seems to bring both accurate predictions and noise to the result (note the drop in precision, as the model will be penalized if it detects a false positive). The better way to incorporate logic rules in applications on large and sparse KGs is left for future work.

**Table 4:** F1 scores on the entire test set.

**Model**	**Micro F1**		
	**P**	**R**	**F1**
Baseline	0.64	0.61	0.634
Ctx Attention	**0.72**	0.56	**0.637**
HRE	0.60	**0.67**	0.634

*The best results are highlighted in bold*.

#### Incorporating New Rules

We also try to incorporate new rules into the induction system. Specifically, we add the meta-rule: $r_{1}=relation\left(s^{*},x\right)\in P^{+}\Rightarrow relation\left(s^{*},o^{*}\right)=r_{2}$. Intuitively, this models the logic that if an object s^*^ has a relation *r*_1_ with another object *x*, then there is an increased probability for another relation *r*_2_ to any other object o^*^. For example, if a man is riding a horse, there is an increased probability that he is wearing a hat. More generally, when an object maintains one relation, it is more likely to maintain further relations. The experimental results showed a large increase in recall but a decrease in precision of the framework. This leads to the conclusion that the logic rules used by the system should be carefully designed based on the underlying application.

### 4.2. Visual Relation Extraction

#### 4.2.1. Entity Pair Encoding in Images

**Figure 7:**
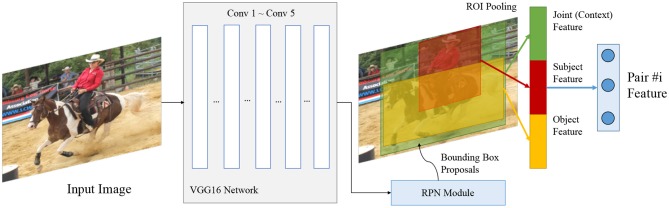
Encoder for visual entity pairs. We extend the union box encoder proposed by Lu et al. (2016) and add the entity's features (what is it) and its location (where is it) into the embedding vector.

Figure 7 illustrates the overall architecture of the visual entity pair encoder. Each object appears as a bounding box in a visual image. The detection, classification, and localization is done with the Faster-RCNN framework (Ren et al., 2015). We extend the method proposed by Lu et al. (2016) to extract the features F(s,o) of the object pair (*s, o*). To obtain the neural relations *rel*_*N*_ we apply a two-layer perceptron followed by a softmax layer on the extracted features F(s,o).

To effectively encode features of an entity pair into distributed representations F(s,o), we extract features of the subject, the object, and their interaction environment. We denote *feat* as the extracted features of the whole input image. These features are extracted with a VGG-16 network pre-trained on MS-COCO (Xu et al., 2017). The features of a given region specified by a bounding box are denoted as *feat*[*box*]. These features are obtained with the Region-Of-Interest (ROI) pooling operation introduced by Girshick (2015). *feat*[*box*_{*s, o*}_] then denotes the features of an individual entity (subject or object), extracted from the image features *feat* at the given bounding box location with the ROI pooling operation.

We model the interaction environment of an entity pair by the union box of their bounding boxes *box*_*s*_, *box*_*o*_. The features of the interaction environment are then denoted as *feat*[*box*_*u*_]. Similar to the marker embedding in textual relation extraction, we specify the locations of subject and object in the interaction environment by adding a mask to the features after ROI pooling. The mask is a binary matrix in the same shape as the feature after ROI pooling of the union box. Each element of the feature after ROI pooling corresponds to a grid region in the original image. Each non-zero element of the mask then corresponds to the Intersection-over-Union (IoU) of the entity bounding box and the bounding box of the bin. Formally, the indices of non-zero elements *Ind*^{*s, o*}^ are given by:

$$Ind_{i,j}^{\left\{s,o\right\}}=IoU\left(Region{\left(box_{u}\right)}_{i,j},box_{\left\{s,o\right\}}\right),$$

where *Region*(*bo*_*x*_*u*_)*i, j*_ is the corresponding region on the image of the grid located at row *i* and column *j* in the ROI Pooling window of *box*_*u*_.

Formally, given the subject features *feat*[*box*_*s*_], object features *feat*[*box*_*o*_], and union features *feat*[*box*_*u*_], the features *F*(*s, o*) of an entity pair are then calculated as follows:

$$\begin{array}{l} F\left(s,o\right)=feat\left[box_{s}\right]\\ \;⊗feat\left[box_{o}\right]\\ \;⊗feat\left[box_{u}\right]\\ \;⊗feat\left[box_{u}\right]⊙{Ind}^{s}\\ \;⊗feat\left[box_{u}\right]⊙{Ind}^{o}, \end{array}$$

where ⊗ is the feature concatenation operation and ⊙ is the element-wise multiplication.

#### 4.2.2. Visual Genome

Visual Genome (Krishna et al., 2016) is a dataset consisting of 108, 077 images. On average, each image contains 21.2 objects and 17.7 relation instances. Due to the poor quality of annotations, we follow Xu et al. (2017) to manually clean up the dataset. We further remove the duplicate relations in each image. The final dataset contains 11.0 distinct objects and 6.0 relation instances per image on average. The average fraction of relations over entity pairs is ~ 6%. We also generate a dense test set which is a subset of the entire test set, where the fraction of relations over entity pairs is at least 15%. The dense test set contains 2, 361 images, with an average of 4.2 distinct objects and 5.3 relations per image.

Following (Lu et al., 2016; Xu et al., 2017) we use Recall@k (R@k) to evaluate models on the task of visual relation extraction. R@k measures the fraction of correct predictions in the top *k* confident predictions. We do not adopt AP (average precision, which can be viewed as the area under precision-recall curve) as our evaluation metric because relations are not exhaustively labeled, as analyzed in Lu et al. (2016).

As shown in Table 5, equipped with a logic deduction module, we gain a significant improvement over the baselines (only *rel*_*N*_) as well as other existing methods. The baseline is identical to the baseline model used in Xu et al. (2017) except the feature extractor. The performance of our baseline model demonstrates the effectiveness of our entity pair embedding.

**Table 5:** Experimental results of visual relation extraction on the entire Visual Genome test set.

	**R@50**	**R@100**
UnionBox	0.279	0.350
MsgPass	0.448	0.531
Baseline	0.489	0.570
HRE	**0.502**	**0.577**

*We compare our model with UnionBox (Lu et al., 2016) and MsgPass (Xu et al., 2017). The best results are highlighted in bold*.

Interestingly, we observe that our model achieves almost identical performance in terms of Recall@k metric on the dense and the entire test set. Since the Recall@k metric does not penalize false positive predictions of the relation, the noise brought by the induction module is significantly reduced compared to the text case.

### 4.3. Implementation Details

For visual relation extraction models, *F*_*entity*_ has 512 channels, and *F*_*union*_ has 256 channels. The window size of ROI Pooling is set to 7 × 7. All fully-connected layers except the ones used by attention model have 4,096 channels following the typical VGG-16 structure. We use a 512-dim vector to represent the attention vector *e*_*i*_.

For textual relation extraction models, we use GloVe50 (Pennington et al., 2014) as the word embedding and 256 as the value for the hidden size of LSTMs and of fully-connected layers.

We implemented the model based on the open-source package PyTorch (Paszke et al., 2017). We optimize the model, including the entity pair encoder and relation predictor, in an end-to-end manner with Adam (Kingma and Ba, 2014) and use cross-entropy loss for the relation classification. The average training time is 0.17 s for a single sentence, and 0.48 s for an image on a GeForce GTX 1080 Ti.

## 5. Conclusion and Future Work

We proposed a novel sequential prediction model for conditional neural relation extraction, which explicitly takes the previously determined or known relations of entity pairs into consideration for better future relation prediction. We achieved this by an induction system based on explanatory logic rules. Experimental results show the superiority of the proposed model in both textual and visual relation prediction tasks. Our model outperforms other existing works when the entity graphs become denser.

An interesting observation of our experiments is that the prediction model shows a stable improvement of performance independent of whether using a textual or visual entity encoder. Since both encoders rely on a high dimensional representation space that inherently encodes the semantic closeness of entities (Lu et al., 2016; Sorokin and Gurevych, 2017), it seems that the relation predictor is in many cases able to derive a prediction for a data point that includes novel or uncommon entities. Similarly to infant learning, the encoders learned the characteristics of entities statistically from the data. As a consequence, this work does not only improve relation extractors but also builds a bridge between brain-inspired neural networks and logic induction systems as well as other KG completion models. For application purposes, the resulting framework is highly customizable and programmable, which opens a new path toward a better machine reasoning system.

Compared to most previous approaches our method can deal better with the long tail in the distribution over relations. Through the use of logic rules and the pair prediction module our approach is able to deal with rare relations and apply them correctly to previously unseen object pairs. This is a key advantage since dealing with the skewed distribution over relations and generalizing relations to unseen object pairs is a key requirement for successful relation extraction from text or images. Furthermore, through the use of differentiable inductive logic our model is trainable in an end-to-end manner, meaning only minimal human involvement and only few hand-crafted rules.

However, the addition of the pair selector increases the size of our model and the number of parameters. Additionally, the rules for the inductive logic still have to be handcrafted and we only evaluated the model with one meta-rule. Future work should, therefore, evaluate how well the approach works with multiple complex logic rules or if it is even possible to learn new, valid rules. Another limitation is that our approach currently only works on either textual or visual relation prediction. In future work, we want to combine textual and visual relation prediction. Our model easily allows to combine multimodal features, e.g., by feeding concatenated visual and textual features to the HRE input (Figure 3, right). This is relevant for human-robot interaction, where dialogue contains not only purely linguistic entities, but where references to entities in the surrounding scene are being made. Aligning textual input, e.g., from transcribed speech, with visual input will enable better linguistic understanding by an embedded agent that matches the verbally perceived relations to the scene, e.g., for disambiguation of an object among others.

## Data Availability Statement

The datasets generated for this study are available on request to the corresponding author.

## Author Contributions

JM and YY contributed to the model design. JM, YY, SH, and TH contributed to the experiments and paper writing. CW, SW, ZL, and MS provided valuable suggestions and helped in revising the paper.

### Conflict of Interest

The authors declare that the research was conducted in the absence of any commercial or financial relationships that could be construed as a potential conflict of interest.

## Footnotes

^1^Image sources. Goose: pngimg.com/download/58532, license CC 4.0 BY-NC. Table: commons.wikimedia.org, author Tangopaso, released into public domain.

^2^Mathematically, given a graph *G* = (*V, E*), the density can be computed as |*E*|/|*V*|^2^.

^3^We normalize the distribution.

^4^A semantic graph can be deduced if it contains at least three connected entities, i.e., at least 1 reasoning chain.
